# Supplementary figures and images for: Nucleolar sequestration of cannabinoid type-2 receptors in triple-negative breast cancer cells
Source: PLoS One. 2025 May 13;20(5):e0323554. doi: 10.1371/journal.pone.0323554 (PMC12074389; doi:10.1371/journal.pone.0323554)

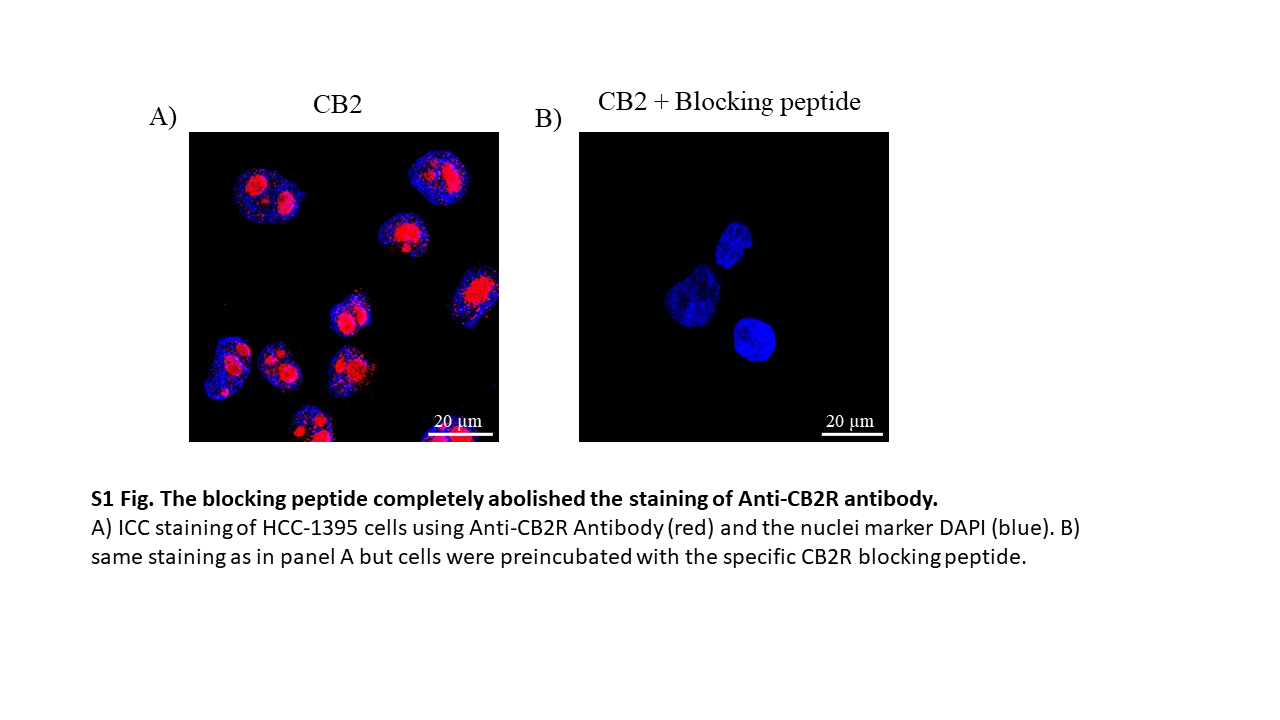

Supplement: S1 Fig — (TIF) [file pone.0323554.s001.tif]
