# Supplementary material for: Nucleolar sequestration of cannabinoid type-2 receptors in triple-negative breast cancer cells
Source: PLoS One. 2025 May 13;20(5):e0323554. doi: 10.1371/journal.pone.0323554 (PMC12074389; doi:10.1371/journal.pone.0323554)
Supplement: Attachment — Submitted filename: Responses to reviewers R1.docx [file pone.0323554.s003.docx]

We sincerely thank the editor and reviewers for the positive constructivism on our study and the suggestions for improvement of our manuscript. Below we paraphrase your specific comments and provide a response in blue font (R). Changes in the manuscript are denoted in red font.

Note: The lines and page numbers indicated here are designated for the Revised Manuscript with Track Changes.

**Responses to Editor's comments:**

All the Editor comments relative to the Journal Requirements have been attended.

**Responses to Reviewers' comments:**

**Reviewer 1:**

1) Include key statistical results, a brief mention of the study's limitations, and the implications for future research in abstract. Ensure the abstract is self-contained with all abbreviations defined at first use.

R. Thank you for your recommendations, which have been added as suggested.

2) Add background of different phenolic compounds in introduction using these articles

R. We included the information of 2 out of the 3 suggested references in the Discussion, because we considered that it was more pertinent in this section (at the end of page 17).

3) Clearly state the research question and hypothesis in the last paragraph of the introduction. Emphasize the novelty of studying CB2R sequestration in TNBC cells.

R. Ok, we addressed these points at the end of Introduction (lines 5-7 and 9-13, page 5).

4) Justify the choice of specific TNBC cell lines (HCC-1395 and MDA-MB-231) and the non-tumoral cell line (MCF-10A). Explain the relevance of these models to the broader field of TNBC research in material and methods.

R. Done (first 4 lines of the *Cell culture and transfection* section in page 5)

5) Provide additional information or references regarding the validation of the anti-CB2R antibody and controls used to confirm specificity in immunocytochemistry.

R. We included a new section in Materials and Methods as *Antibody Characterization* (page 7) and included a new figure (S1 Fig) where the specificity of the CB2R antibody was tested against its blocking peptide.

6) Discuss any limitations or potential biases in image acquisition and analysis, such as variations in staining intensity or observer bias.

R. We added a few lines about these issues almost at the end of the Image Acquisition and Analysis section (lines 9-12, page 8).

7) Integrate the findings of this study with existing literature in discussion. Expand the discussion to include a comparison with previous studies on CB2R in other cancer types. Address the broader implications of nucleolar CB2R localization for cancer therapy.

R. The main and novel contribution of our work is that TNBC cells exhibit a high accumulation of CB2R in the nucleolus, and thus there is no precedent of nucleolar sequestration of CB2R not only in cancerous cells but in any other type of cell. Hence, we could not include a comparison with previous studies on CB2R in other cancer types. For this same reason, we could not speculate the implication of our finding in the cancer therapy, but only claiming its potential value as a TNBC biomarker (as commented at the end of the Abstract, Introduction and Discussion).

8) Suggest specific next steps for future research in conclusion. Avoid overgeneralizing the implications of the study.

R. Done (at the end of the Discussion in page 18)

9) Use up-to-date references; particularly studies published in the last two years.

R. We already backed up our information with more up-to-date references by replacing some of them or adding another ones.

**Reviewer 2:**

1) Write the statistical significance p-value in the abstract as well.

R. Done

2) Statistical Analysis needs attentions.

R. In this resubmission we have added the statistical significance both in the Abstract and Results sections, additionally to what was previously mentioned in the Statistical Analysis section regarding the tests for assessing the data distribution (Shapiro-Wilk and Kolmogorov-Smirnov), and thus to apply either parametric (unpaired *t*) or non-parametric tests (Mann-Whitney U or Kruskal-Wallis). In Figure legends, it is indicated the specific test or post hoc test and the statistical significance corresponding to the panels.

3) You can suggest these papers and can add them as related to breast cancer cell lines http://www.pvj.com.pk/pdf-files/24-218.pdf

https://vetdergikafkas.org/abstract.php?lang=en&id=3068

R. Ok, we enriched the Discussion by including one of the suggested references (at the end of page 17).

4) Mention the magnification power of the confocal microscope used to capture the breast cells.

R. In the Image Acquisition section, we have clarified that images where obtained using only 40× or 63× objectives, with no optics zoom (line 3, page 8).

**Reviewer 3:**

I have reviewed the manuscript and am pleased to accept it for publication. The work is soundly written, presents a significant contribution to the field, and adheres to the journal's standards. The research is well-executed, and the findings are both novel and relevant. The manuscript is clear, logically structured, and effectively communicates its findings. I believe it will be a valuable addition to the literature.

R. Thank you for your comments on our work, we appreciate the time spent on this review.
