# Supplementary material for: Nucleolar sequestration of cannabinoid type-2 receptors in triple-negative breast cancer cells
Source: PLoS One. 2025 May 13;20(5):e0323554. doi: 10.1371/journal.pone.0323554 (PMC12074389; doi:10.1371/journal.pone.0323554)
Supplement: Attachment — Submitted filename: Response to Editor.docx [file pone.0323554.s005.docx]

**RESPONSES TO THE EDITOR’S COMMENTS**

We thank to the editor for his comments on our study and the suggestions to improve our MS. As in the preceding resubmission, changes in the MS are shown in red font, while responses (R) to your specific comments are denoted in blue font.

1) The keywords should not be included in the title. Please revise accordingly.

R. Done

2) The beginning of the second paragraph of introduction is not appropriate. Consider rewording for better clarity and coherence.

R. Done

3) Improve the overall English language and revise for better readability.

R. Done

4) Check for any grammatical mistakes and correct them.

R. We have already revised and corrected the grammar of the manuscript in more detail.
